# Supplementary material for: Dependence of Leydig Cell’s Mitochondrial Physiology on Luteinizing Hormone Signaling
Source: Life (Basel). 2020 Dec 31;11(1):19. doi: 10.3390/life11010019 (PMC7824612; doi:10.3390/life11010019)
Supplement: Supplementary file 1 [file life-11-00019-s001.pdf]

Supplemental Table A1. Primers Sequence

Gene	***Gene Accession number***	***Primer Sequence***
B2m	NM_012512.2	F: 5'-GCGTGGGACGAGCATCAGGG -3'<br>R: 5'-CTCATCACCACCCCGGGGACT -3'
Cox4/2	NM_053472	F: 5'-CACAGCCCAGGAAGTGCTGCTA-3'<br>R: 5'-TGTGCAGTAAGGCTCATCCGGC-3'
Cyp11a1	NM_017286.2	F: 5'-CAAAACACCACGCACTTCC-3'<br>R: 5'-TCAATTCTGAAGTTTTCCAGCA-3'
Cytc	NM_012839	F: 5'-GCAAGCATAAGACTGGACCAAA-3'<br>R: 5'-TTGTTGGCATCTGTGTAAGAGAATC-3'
Drp1	NM_053655.3	F: 5'-AGCAGAAGAATGGGGTAAATTTCTT -3'<br>R: 5'- GCTCAGGGCTTACTCCCTTATT -3'
Fis1	NM_001105919.1	F: 5'-ACGCCTGCCGTTACTTCTTC-3'<br>R: 5'-GCAACCCTGCAATCCTTCAC-3'
Gapdh	NM_017008	F: 5'-TGCCAAGTATGATGACATCAAGAAG-3'<br>R: 5'-AGCCCAGGATGCCCTTTAGT-3'
Mfn1	NM_138976.1	F: 5'-CCTTGTACATCGATTCCTGGGTTC-3'<br>R: 5'-CCTGGGCTGCATTATCTGGTG-3'
Mfn2	XM_008764288.2	F: 5'-TCAAGCGCCAGTTTGTGGAG-3'<br>R: 5'-CACAGATGAGCAAATGTCCCAGA-3'
mtNd1	NC_001665.2:2740-3694	F: 5'-GCGTGGGAGGAGCATCAGGG-3'<br>R:5'-GCGAATGGTCCTGCGGCGT-3'
Nrf1	NM_001100708	F: 5'-GACCATCCAGACGACGCAAGCA-3'<br>R: 5'-ATGGGCGGCAGCTTCACTGTT-3'
Nrf2a	NM_001108841	F: 5'-AGCGGAACTGAACCGCTTGGT-3'<br>R: 5'-GTGACTGGCTGAGCAATCCCGT-3'
Opa1	NM_133585.3	F: 5'-AAAAGCCCTTCCCAGTTCAGA-3'<br>R: 5'-TACCCGCAGTGAAGAAATCCTT-3'
Pink1	NM_001106694.1	F: 5' CAAGCAAGTGTCTGACCCAC 3'<br>R: 5' GCTTCATACACAGCGGCATT 3'
Pprgc1a	NM_031347	F: 5'-AGCCGTAGGCCCAGGTATGACA-3'<br>R: 5'-TGCTTGGCCCTTTCAGACTCCC-3'
Prkn	NM_020093.1	F: 5' CTTCCAGCTCAAGGAAGTGG 3'<br>R: 5' CAGAGGCATTTGTTTCGTGA 3'

Star	NM_031558	F: 5’-AGCCAGCAGGAGAATGGAGAT-3’<br>R: 5’-CACCTCCAGTCGGAACACCTT-3’
Tfam	NM_031326	F: 5'-TATAGTCGTCGGCCCGAGGGAT-3'<br>R: 5'-AAGGCTGACAGGCGAGGGTATG-3'
Tfeb	NM_001025707.1	F: 5'-CGACAACATTATGCGCCTGG-3'<br>R: 5'-CTGTACACGTTCAGGTGGCT-3'
Ucp2	NM_019354	F:5'-ACGACCTCCCTTGCCACTTCAC-3'<br>R: 5'-GGTACTGGCCCAAGGCAGAGTT-3'
